# High-Transmittance Subwavelength Metal Grating with Relief Structure Composed of Multiple Steps

**DOI:** 10.1155/2014/495820

**Published:** 2014-02-18

**Authors:** Zhongfei Wang, Dawei Zhang, Qi Wang, Banglian Xu, Qingyong Tang, Yuanshen Huang, Songlin Zhuang

**Affiliations:** Shanghai Key Laboratory of Modern Optical System, Optical Instruments and Systems Engineering Research Center of Ministry of Education, School of Optical-Electrical and Computer Engineering, University of Shanghai for Science and Technology, Shanghai 200093, China

## Abstract

A new kind of subwavelength metal grating with relief structure is designed and analyzed, in which the shape of the grating lines is no longer a single rectangle, but a relief structure with multiple steps. GsolverV52 was used to determine the optimal values of the grating period, groove depth, and the number of steps. The optical performance of the novel structure is evaluated and compared in terms of the transmission efficiency and extinction ratio over the visible and near-infrared wavelength spectrum. It is shown that, in the near-infrared band, the maximum transmittance can be increased about 15% compared to the traditional metal grating under the same parameters. With the unique characteristics, the metal grating is expected to find applications in liquid crystal display fields, polarization imaging, optical communication, and so on.

## 1. Introduction

As an indispensable optical element, gratings play the pivotal role in all kinds of optical systems. Since Nevdakh et al. [1] had studied the polarization characteristics of grating the subwavelength grating has become the focus of researches in recent years for it possesses the characteristics of having great polarization properties and small volume and being easy to be integrated. Theories and experiments all show that when the period of the grating is close to or smaller than the wavelength of the incident light it always performs strong polarization characteristics. With this feature, we can produce a variety of polarizing devices, such as various wave plates [2], polarizing beam splitters [3–7], and polarizing color filters [8, 9]. On the other hand, the fabrication has been very mature. It could be manufactured on different materials such as soft-composite materials which could find attractive applications in LCD [10, 11]. Diffraction grating also is used in optical communication, polarizing LED [12, 13], polarization imaging [14], and other fields.

According to the grating equation, when light propagates onto the subwavelength grating surface, only the 0th-order diffraction exists [15]. Traditional subwavelength metal grating transmittance is generally about 70% [16] in visible region and about 90% in near-infrared region [17]. Obtaining high transmittance and great extinction ratio is the goal of the researchers. One way is to reduce the grating period. The studies found that the smaller the period, the higher the transmittance for subwavelength metal grating. However, the difficulty of manufacture and the cost also relatively increased. Professors try to find another way that is changing the structures of gratings, for example, adding a high refractive-index dielectric layer [16] and using a periodic array of annular apertures [18]. In the past few years, Zhou et al. [19] proposed a subwavelength metal grating with double metal layers, while increasing the transmittance as well as extinction ratio, and obtained a more uniform transmission spectrum intensity. Wu et al. [6] studied a double-groove grating which achieved high transmittance and extinction ratio for TM-polarization as well as high reflectivity and extinction ratio for TE-polarization, which can be used as a polarizing beam splitter. By integrating a nearby metallic nanostrip over the nanoslit opening, Cui and He [20] introduced a resonant nanocavity antenna to couple more incident light to enhance the transmission.

In this paper, we propose a novel subwavelength metal grating with relief structure of the grating lines composed of multiple steps. The steps increased by one layer to four; simultaneously, the duty cycles of each layer changed regularity. Optimized by use of GSolverV52 around the wavelength range of 400 nm–2500 nm, we attempt to explore the influences on transmittance and extinction ratio caused by the changes of steps number and groove depth.

## 2. Design and Theoretical Analysis

**Figure 1:**
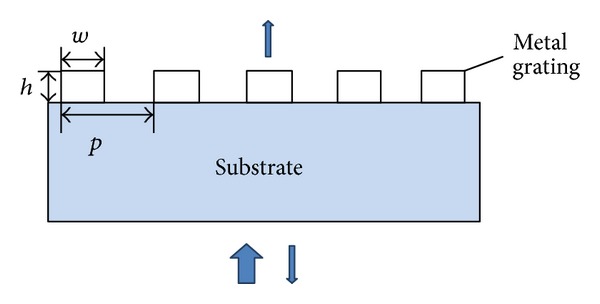
Schematic of traditional subwavelength metal grating.

**Figure 2:**
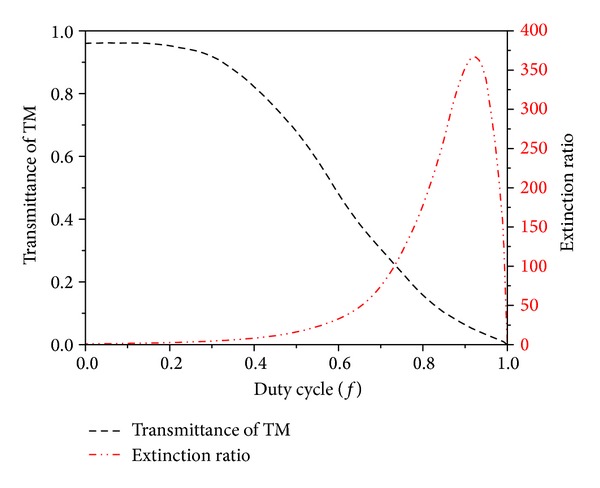
Calculated relationship between the duty cycle and the transmittance of TM and extinction ratio.

Figure 1 illustrates the traditional subwavelength metal grating structure, namely, wire grids uniformly distributed on the transparent substrate. The mechanism of the unique polarization characteristic is that TE polarized light could stimulate current formed by the nanowires electronics, which allowed the light along this direction reflected back. However, for TM polarized light, for the existence of the air gap between the nanowires, it can just be transmitted [19]. If *h* is the groove depth, *p* is the grating period, and *w* is the width of ridge, that *f* = *w*/*p* is the corresponding duty cycle. It plays an important role in determination of the optical properties of the grating. For example, the properties and wavelength band of polarization-independent grating depend strongly on the grating duty cycles [21]. In fact the slit in the grating structure can be considered as an F-P cavity that has two mirrors of finite reflectivity at both ends of the slit [22]. Owing to different metal materials with different transmission rate and extinction ratio, thus choosing the right metal is the primary problem. Combining with microprocessing capacity at present and the characteristic curves of aluminum, silver, gold, and chromium, the basic parameters of the proposed grating are *p* = 200 nm, the material aluminum and the refraction index of substrate is 1.5. As *h* = 60 nm and the incident wavelength is 550 nm, the transmittance of TM-polarization and extinction ratio as a function of the duty cycles for the optimized metal grating are shown in Figure 2.

From Figure 2, we can find that, with the increase of duty cycles, the transmittance of TM-polarization decreased until zero, while the extinction ratio increased first and then decreased rapidly. Therefore, the transmittance and extinction ratio of the metal grating fixed period and groove depth is a pair of contradiction as a function of duty cycle. It means we cannot improve the extinction ratio and transmittance simultaneously. That is to say, small transmission efficiency of TM-polarization will make large extinction ratio and large transmission efficiency of TM-polarization will get small extinction ratio. Therefore, the duty ratio of the grating must be determined according to the actual demands.

**Figure 3:**
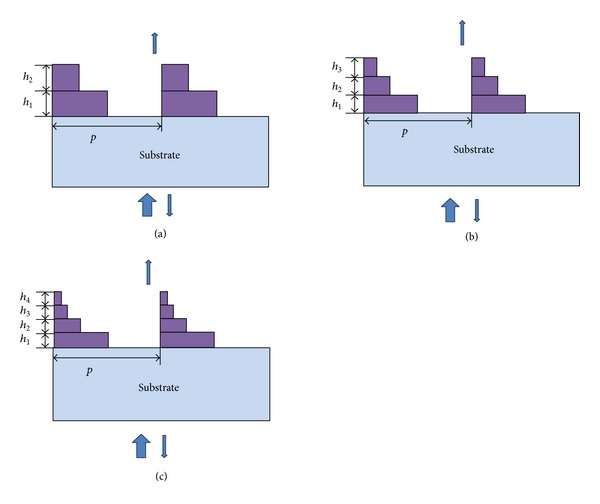
Schematic of subwavelength metal grating with relief structure composed of multiple.

In order to improve the optical performance of conventional single layer gratings, subwavelength metal gratings with relief structure made by multiple steps are showed in Figure 3. We can see that the steps change from one to four, and total height is *h*. In the simulation process, we should ensure that the height of each layer is the same, that is, the total height distributed averagely, where heights are *h*_1_, *h*_2_, *h*_3_, *h*_4_ and corresponding duty cycles are 0.0625, 0.125, 0.25, and 0.5. According to [6, 8], the subwavelength metal grating has large angular tolerance; that is to say, angle changes cause little influence on transmittance. Thus, a monochromatic plane wave is incident from the air with an incident angle *θ* = 0.

## 3. Results and Discussion

**Figure 4:**
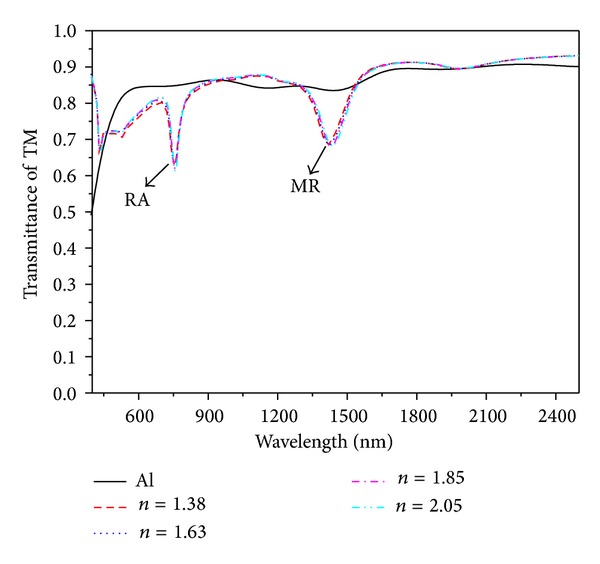
Calculated relationship between refractive index of middle layer and the transmittance of TM.

The impact aroused by the high refractive-index dielectric layer must be taken into account. A case study of three-layer structure, replaces the middle tier with high refractive index medium layers and refractive indexes 1.38, 1.63, 1.85, and 2.03, respectively, the results as shown in Figure 4. Obviously the unshifted dips, located around 760 nm and 1425 nm, according to literature 23, are related to the Rayleigh anomaly (RA) on the metal/substrate and localized magnetic resonance (MR) related to the metal/substrate/metal sandwich, respectively [23]. Compared to the original curve, for our experiment, the high refractive index medium layer effect is not ideal.

**Figure 5:**
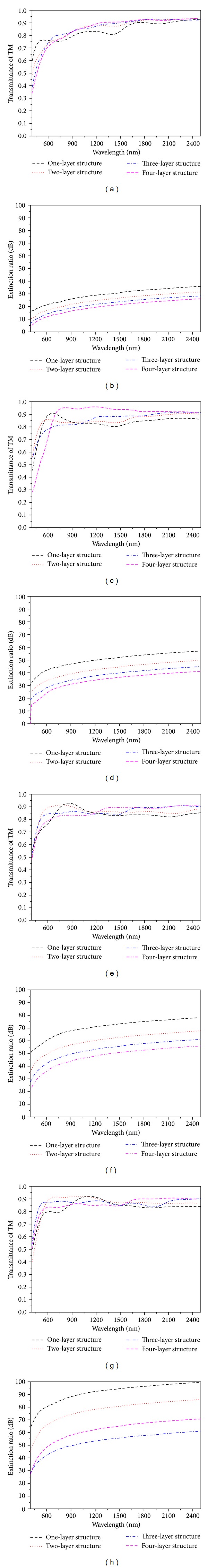
Relationship between groove depth and steps and the transmittance and extinction ratio as a function of wavelength ((a), (c), (e), and (g) stand for the transmittance of TM-polarization; (b), (d), (f), and (h) stand for extinction ratio).

For the groove depth an important parameter also has a great effect on the optical properties of metal grating. In the case of different groove depth and different step number it is necessary to discuss the transmittance of TM-polarization and extinction ratio as a function of wavelength. The groove depths are 100 nm, 200 nm, 300 nm, and 400 nm, and step numbers are from one to four corresponding duty cycles as above mentioned, and the incident wavelength is from 400 nm to 2500 nm. The simulation results were showed in Figure 5.

Compared with the simulation results in Figure 5, we can draw the conclusion that when the groove depth *h* = 200 nm, the effect of improving transmittance is the most obvious. In visible region, the transmittance decreases along with steps increased. However, it increased clearly especially during 1000 nm–1500 nm; the numerical value could have reached 15%, that is, from 80% to 95%. Of course, the extinction ratio was also decreased with the increasing of steps in the whole band. In the near-infrared region it meets the requirements compared with the currently accepted extinction ratio threshold *C* = 20 dB [14]. When groove depth is the other numerical values, we also can get similar results although not ideal.

## 4. Conclusions

In this study we have designed and analyzed the subwavelength metal grating with relief structure, which really has an effect on the aspect of improving TM-polarization transmittance compared to traditional metal grating. The parameters of grating (i.e., the period *p*, grating groove depth *h*, and steps) have been optimized with the help of GSolverV52. The best results have shown that about 15% was improved corresponding to the extinction ratio greater than 20 dB in near-infrared region while *p* = 200 nm and *h* = 200 nm. The idea of proposed grating can be considered as a useful method to improve the optical performance although the fabrication is complex. It opens up a new dimension in the design and operation of optical systems.
